# Autoimmune Idiopathic Inflammatory Myopathies: Pharmacological Differences and Similarities by Type of Myositis and by Sociodemographic Variables

**DOI:** 10.1155/2022/1807571

**Published:** 2022-07-05

**Authors:** Luis Fernando Valladales-Restrepo, Ana Camila Delgado-Araujo, Brayan Stiven Aristizábal-Carmona, Lina María Saldarriaga-Rivera, Jorge Enrique Machado-Alba

**Affiliations:** ^1^Grupo de Investigación en Farmacoepidemiología y Farmacovigilancia, Universidad Tecnológica de Pereira-Audifarma S. A, Pereira, Risaralda, Colombia; ^2^Grupo de Investigación Biomedicina, Facultad de Medicina, Fundación Universitaria Autónoma de las Américas, Pereira, Colombia; ^3^Semillero de Investigación en Farmacología Geriátrica, Facultad de Medicina, Fundación Universitaria Autónoma de las Américas, Pereira, Risaralda, Colombia; ^4^Grupo de Investigación en Medicina Interna, Universidad Tecnológica de Pereira, Pereira, Risaralda, Colombia

## Abstract

**Objective:**

Autoimmune idiopathic inflammatory myopathies (IIMs) are a group of pathologies that are generally characterized by muscle weakness. Their treatment involves glucocorticoids and immunosuppressants. The aim was to identify differences and similarities in the pharmacological management of a group of patients with autoimmune IIMs according to the type of disease, sex, age group, and city of residence in Colombia from 2020 to 2021.

**Methods:**

This cross-sectional study identified medication prescription patterns for outpatient use in patients with autoimmune IIMs between 2020 and 2021 based on a population database of 8.5 million Colombians affiliated with the Colombian health system. Sociodemographic and pharmacological variables were considered.

**Results:**

A total of 671 patients with autoimmune IIMs were identified, with a median age of 57 years, and 70.9% were women. Overlap myositis was the most frequent disease (31.4%). A total of 91.5% of the patients received pharmacological treatment, mainly systemic glucocorticoids (78.5%), conventional disease-modifying antirheumatic drugs (DMARDs) (74.1%), immunosuppressants (9.1%), and biological DMARDs (3.7%). Pharmacological management predominated among patients with overlap myositis, those who lived in cities, and those affiliated with the contributory regime of the Colombian health system. Conventional DMARDs were prescribed mainly to women and to those older than 65 years.

**Conclusions:**

Patients with autoimmune IIMs are not treated homogeneously. The pattern of drug use varies according to the type of IIM, sex, age group, city, and health system regime affiliation.

## 1. Introduction

Autoimmune idiopathic inflammatory myopathies (IIMs) are a group of rare immune-mediated, multisystemic, heterogeneous diseases that mainly affect skeletal muscle and the skin but can also affect many other organs, such as the lungs, heart, joints, and gastrointestinal tract [1–3]. They are mainly characterized by progressive, symmetrical muscle weakness, and sometimes myalgias, but in addition, heliotrope erythema, Gottron papules, and cutaneous ulcers may appear on the skin. Extramuscular manifestations may also emerge, such as fever, arthralgia, Raynaud's phenomenon, arrhythmias, and dysfunction. Ventricular and pulmonary complications are mainly due to interstitial lung disease [4–6]. The prevalence varies between 2.4 and 33.8 per 100,000 inhabitants, and the incidence ranges from 1.16 to 19 per million people per year [7]. In Colombia, the estimated global prevalence is 25.7 cases per 100,000 inhabitants [8].

IIMs traditionally include polymyositis, dermatomyositis, juvenile dermatomyositis, inclusion body myositis, immune-mediated necrotizing myopathy, and antisynthetase syndrome [1, 3, 9]. In addition, inflammation of the skeletal tissue can occur in the context of other connective tissue diseases, such as systemic lupus erythematosus, rheumatoid arthritis, Sjögren's syndrome, and systemic sclerosis, a condition called overlap myositis [3, 5]. Its management seeks to control the inflammatory process and prevent damage to skeletal muscle or extramuscular organs [2]. Depending on the type of autoimmune IIM and its severity and complications, systemic glucocorticoids, conventional disease-modifying antirheumatic drugs (DMARDs) (especially azathioprine or methotrexate), or immunosuppressants (primarily cyclophosphamide, cyclosporine, human mycophenolate mofetil, or gamma-globulin) are prescribed. Biological DMARDs (mainly rituximab) [2–4, 6, 10] are given, but the response to treatment varies [3].

The Colombian health system offers universal coverage to the entire population through two regimes, including one contributory (paid by the worker and employer) and another subsidized by the state, and has a benefit plan that includes a heterogeneous group of medications used for the treatment of autoimmune IIMs. Sociodemographic factors such as age, sex, location of residence, and type of health system coverage can influence the use of medications [11–13], as well as the type of autoimmune IIM diagnosed [14]. Therefore, we aimed to identify differences and similarities in the pharmacological management of a group of Colombian patients with autoimmune IIMs according to the type of disease, sex, age group, place of residence (capital city vs. smaller city), and system regime affiliation in 2020-2021.

## 2. Materials and Methods

An observational cross-sectional study was conducted on the prescription patterns of drugs used in patients diagnosed with autoimmune IIMs based on a population database that collects information from approximately 8.5 million people affiliated with the Colombian health system through six health insurance companies, corresponding to approximately 30.0% of the active affiliated population of the contributory or payment regime and 6.0% of the state-subsidized regime, accounting for 17.3% of the Colombian population.

Patients were identified and classified using International Classification of Diseases (ICD-10) codes, including those for juvenile dermatomyositis (M330), dermatomyositis (M331), polymyositis (M332), and dermatopolymyositis (M339) in the period between January 1, 2020, and December 31, 2021. Patients with a concomitant diagnosis of rheumatoid arthritis (M053, M058-M060, M068, M069, and M080), systemic sclerosis (M340, M348, and M349), systemic lupus erythematosus (M321, M328, and M329), and Sjögren's syndrome (M350) were considered to have overlap myositis. Patients of any age and sex who attended outpatient medical consultations were selected. Those with two or more different diagnoses of autoimmune IIMs and those who appeared only once with a considered diagnosis in the study period were excluded.

Based on information on drug consumption for the affiliated population systematically obtained from the dispensing company (Audifarma S.A.), a database was designed in which we gathered the following groups of patient variables:

(1) Sociodemographic: sex, age (<40 years, 40-64 years, and ≥65 years), regime affiliation (contributory or subsidized), and dispensation city. The place of residence was categorized into a region according to the classification of the National Administrative Department of Statistics (DANE) of Colombia (the entity responsible for the planning, processing, analysis, and dissemination of official statistics in Colombia) as follows: Bogotá-Cundinamarca region, Caribbean region, Central region, Eastern region, Pacific region, and Amazonia-Orinoquía region. The city of residence was classified as a capital city or an intermediate municipality
(2) Comorbidities were identified from the diagnoses reported by the ICD-10 in the selected patients
(3) Medications:
  - Systemic glucocorticoids: prednisolone, prednisone, deflazacort, methylprednisolone, dexamethasone, hydrocortisone, and betamethasone
  - Conventional DMARDs: methotrexate and azathioprine. Others: chloroquine, hydroxychloroquine, leflunomide, and sulfasalazine
  - Immunosuppressants: mycophenolate mofetil, cyclophosphamide, cyclosporine, tacrolimus, and human gamma globulin
  - Biological DMARDs: rituximab. Others: infliximab, etanercept, adalimumab, and certolizumab
(4) Comedications were grouped into the following categories: (a) antidiabetics (oral and subcutaneous), (b) antihypertensives and diuretics, (c) lipid-lowering drugs, (d) antiulcer drugs, (e) antidepressants, (f) anxiolytics and hypnotics (benzodiazepines and Z drugs), (g) thyroid hormone, (h) antipsychotics (typical and atypical), (i) antiepileptics, (j) antiarrhythmics, (k) antihistamines, (l) antidementia drugs, (m) analgesics (acetaminophen and opioids), (n) nonsteroidal anti-inflammatory drugs, and (o) inhaled bronchodilators and corticosteroids and others

The protocol was approved by the Bioethics Committee of the Universidad Tecnologica de Pereira in the category of research without risk. The ethical principles established by the Declaration of Helsinki were respected.

The data were analyzed with the statistical package SPSS Statistics, version 26.0 for Windows (IBM, USA). A descriptive analysis was performed with frequencies and proportions for the qualitative variables and measures of central tendency and dispersion for the quantitative variables depending on their parametric behavior established by the Kolmogorov–Smirnov test. Quantitative variables were compared by Student's *t*-test or the Mann–Whitney *U* test, and categorical variables were compared by the *χ*^2^ test or Fisher's exact test. Statistical significance was accepted at *p* < 0.05.

## 3. Results

A total of 671 patients diagnosed with some autoimmune IIMs were identified, who were distributed in 71 different cities or municipalities. The percentage of women was 70.9% (*n* = 476). The median age was 57.0 years (interquartile range: 43.0-66.0 years; range: 19.0-93.0 years), and the patients were distributed in the following age groups: <40 years (*n* = 135; 20.1%), 40-64 years (*n* = 346; 51.6%), and ≥65 years (*n* = 190; 28.3%). Most of them lived in the Bogotá-Cundinamarca region (*n* = 240; 35.8%), followed by the Caribbean region (*n* = 141, 21.0%), the central region (*n* = 130; 19.4%), the Pacific region (*n* = 130; 19.4%), and the eastern Amazon region (*n* = 30; 4.5%). Three-fourths (*n* = 508; 75.7%) of them took their medications in capital cities. A total of 87.9% (*n* = 590) were affiliated with the contributory regime, and 12.1% (*n* = 81) were affiliated with the country's subsidized health system.

Most patients had a diagnosis of overlap myositis (*n* = 211; 31.4%), followed by polymyositis (*n* = 198; 29.5%), other dermatomyositis (*n* = 145; 21.6%), dermatopolymyositis (*n* = 113; 16.8%), and juvenile dermatomyositis (*n* = 4; 0.6%). Among the most frequent comorbidities in this group of patients were arterial hypertension (*n* = 249; 37.1%), diabetes mellitus (*n* = 139; 20.7%), and hypothyroidism (*n* = 124; 18.5%). According to groups, rheumatological pathologies (*n* = 274; 40.8%) were the most prevalent, followed by endocrine (*n* = 261; 38.9%) and cardiovascular pathologies (*n* = 252; 37.6%). Among the patients with overlap myositis, the most frequently noted concomitant rheumatological diseases were rheumatoid arthritis (*n* = 119/211; 56.4%), systemic lupus erythematosus (*n* = 64; 30.3%), Sjögren's syndrome (*n* = 55; 26.1%), and systemic sclerosis (*n* = 13; 6.2%). A total of 23.4% (*n* = 157) had some infection and predominantly urinary tract infections (*n* = 61; 9.1%), followed by upper respiratory tract infections (*n* = 43; 6.4%), skin infections (*n* = 26; 3.9%), intestinal infections (*n* = 22; 3.3%), and lower respiratory tract infections (*n* = 20; 3.0%).

A total of 91.5% (*n* = 614) of patients received pharmacological treatment for autoimmune IIMs, especially systemic glucocorticoids (*n* = 527; 78.5%), particularly prednisolone (*n* = 414; 61.7%) and prednisone (*n* = 149; 22.2%), followed by conventional DMARDs (*n* = 497; 74.1%), with prescriptions for azathioprine (*n* = 327; 48.7%) and methotrexate (*n* = 242; 36.1%) predominating, while the use of immunosuppressants was found in 9.1% (*n* = 61) of patients, and biological DMARDs were used by 3.7% (*n* = 25). The main comedications identified in this group of patients were analgesics (*n* = 449; 66.9%), antiulcer agents (*n* = 413; 61.5%), antihypertensives/diuretics (*n* = 288; 42.9%), nonsteroidal anti-inflammatories (*n* = 283; 42.2%), and antihistamines (*n* = 242; 36.1%).

### 3.1. Associations between the Type of Autoimmune Idiopathic Inflammatory Myositis and Some Sociodemographic Variables

Systemic glucocorticoids, immunosuppressants, and conventional and biological DMARDs predominated in a statistically significant manner in overlap myositis (Table 1 and Supplementary Table 1). Prednisolone, prednisone, and conventional DMARDs were prescribed significantly more frequently to women (Table 2). With respect to age, conventional DMARDs were used more often in adults older than 65 years, but chloroquine predominated among those younger than 65 years (Table 3). Significant differences were found between the place of origin and the type of health system regime affiliation, where pharmacological treatment and the use of conventional DMARDs predominated among patients from capital cities and among those affiliated with the contributory regime (Tables 4 and 5, respectively).

## 4. Discussion

This study allowed us to identify the pattern of prescription medications taken by patients with autoimmune IIMs as evidence of the use of medications in the real world in a group of people affiliated with the Colombian health system. To the best of our knowledge, this is the largest study of patients with these pathologies in Colombia or Latin America. The median age of the patients was higher than that found in other studies (34.3-52.5 years) [9, 14–18], although a predominance of women was found in all such studies (63.3-69.0-82.7%) [9, 14–20]. On the other hand, the characterization of the main comorbidities was also consistent with that found in other publications [12, 14, 17, 18, 21].

In this analysis, most patients were diagnosed with overlap myositis, which is consistent with observations reported by Chinniah and Mody in a cohort from South Africa (39.4%) [19] but is not consistent with observations found in the European registry of inflammatory myopathies (EuroMyositis Registry), where dermatomyositis predominated (31.0%) [9], as in Asia (42.0-63.3%) [15, 16, 22] and South America (43.9-62.9%) [17, 21], while in Spain, cases of polymyositis prevailed (29.0-40.1%) [14, 18]. These differences may be methodological in nature, deriving from the type of study, the inclusion and exclusion criteria, the method of identifying the patients, the source of information, the diagnostic criteria used, and the period during which the cases were identified, as well as the different geographical regions where the research was conducted [8, 9, 14–19, 21, 22]. In this study, the patients were identified by their ICD-10 codes, but the ICD-10 does not have an exact diagnosis for some autoimmune IIMs, such as antisynthetase syndrome, immune-mediated necrotizing myopathy, and inclusion body myositis [23], which leads to the available codes being used to cover different types of myositis [3].

Most patients received some medication for their autoimmune IIMs in contrast to data found in the EuroMyositis Registry, where only one-third of patients were receiving treatment at the time of publication [9]. The proportion of patients with glucocorticoid prescriptions was very similar to that found by Salazar et al. in two high-complexity institutions in Colombia (81.3%) [17] and by Smoyer et al. in the USA (72.7%) [12] but was higher than that found in the EuroMyositis Registry (31.6%) [9] and in the last consultation of the Myopathies Registry of the Community of Madrid (REMICAM Cohort) (56.6%) [14]. Among the conventional DMARDs, azathioprine and methotrexate were the most commonly used, which is consistent with other reports [9, 14–18, 22]. Among the biological DMARDs, rituximab was the most commonly used, which is also consistent with the literature [9, 12, 14, 17, 18, 22]. On the other hand, among the immunosuppressants, a predominance of mycophenolate mofetil was found, which is consistent with findings in India [22] and the USA [12] but not with findings in other countries, where cyclophosphamide prevailed [14, 15, 17, 18]. The differences in drug use patterns may be due to the characteristics of health systems, the accessibility and availability of drugs in each country, the management guidelines followed, the preferences of the prescriber, the marketing strategies of the pharmaceutical industry, the disease severity and complications, the type of myopathy, the disease course, and the patient tolerability to these drugs [6, 11, 24].

In general, most patients with autoimmune IIMs were treated with the medications indicated by guidelines [3–6, 10], but notably, the management of IIMs is challenging due to the heterogeneous behavior of the different entities and the absence of multidisciplinary and comprehensive management guidelines [3, 6] and evidence-based recommendations for the management of patients with extramuscular conditions, comorbidities, and severe manifestations [6]. In this study, differences were found in the pattern of drug use according to the type of autoimmune IIM, which is consistent with other reports [9, 17, 22]. The predominance of different therapeutic groups among patients with overlap myositis is notable, as described in Spain, where Nuño-Nuño et al. found that these patients had more prescriptions for glucocorticoids, methotrexate, mycophenolate, and cyclophosphamide than those who diagnosed with dermatomyositis or polymyositis [14]. In China, Xiao et al. found sociodemographic, clinical, and paraclinical differences between these patients, but their pharmacological treatments were not evaluated [25]. The greater use of medications in this group of patients is due to the concomitant presence of other connective tissue diseases [14, 15, 21, 26, 27]. On the other hand, patients with inclusion body myositis do not usually respond to the therapies recommended for other autoimmune IIMs [3–5, 10]. However, these cases could not be identified due to the methodological limitations of our study.

Drug prescriptions were not homogeneous with respect to certain sociodemographic variables. Prednisolone/prednisone and conventional DMARDs prevailed among women. Such differences between sexes have also been documented in studies involving other rheumatological diseases [11, 28, 29]. Thus, among patients with systemic lupus erythematosus, glucocorticoids, immunosuppressants, chloroquine, and azathioprine predominate for men [11]. In patients with axial spondyloarthropathies, prednisone and conventional DMARDs prevail for women [28], and among patients with ankylosing spondylitis, glucocorticoids and methotrexate predominate for women, while biological DMARDs predominate for men [29]. These differences in treatment, rather than being due to health inequalities due to sex, are better explained by genetic and hormonal differences between men and women, the greater burden of autoimmune morbidity in women—which affects the degree of activity, the progression, the severity, and the prognosis of rheumatological diseases—and the effectiveness of pharmacological therapy [11, 28–30].

In general, treatment with conventional DMARDs strongly predominated among older adults, which differs from observations in patients with systemic lupus erythematosus, where pharmacological therapy with conventional DMARDs, glucocorticoids, and immunosuppressants has decreased with increasing age [11], while in patients with rheumatoid arthritis, treatment with glucocorticoids was similar between all age groups, and biological DMARDs were more likely to be used by younger patients [31]. Differences were also found in pharmacological management according to whether the patients lived in a capital city or municipality. In the USA, Deodhar et al. found that variations among patients with ankylosing spondylitis depend on the geographic region of care [32]. Similarly, in Colombia, other pharmacoepidemiological studies involving antirheumatic drugs and other therapeutic groups have shown differences in the pattern of prescription to patients [11, 13], which might be due to differences in access to the health system, resource availability, and quality of care [33].

Most patients were affiliated with the contributory regimen, which is consistent with an earlier study in Colombia [8]. In this report, some differences were found in the management received according to the type of health system regime affiliation, which is in line with a USA study, where the databases of three health insurance policies were compared (commercial and Medicare patients vs. Medicaid patients), revealing that in general, commercial and Medicare patients received more medications, especially systemic glucocorticoids and methotrexate, than Medicaid patients [12]. In addition, among patients with psoriatic arthritis and ankylosing spondylitis under the same health policy, greater use of conventional and biological DMARDs was found for Medicare patients [34]. Similarly, in Argentina, in patients with systemic lupus erythematosus, the authors found that cyclophosphamide was significantly more commonly used in the public sector than in the private sector [35].

Some limitations might complicate interpretation of our results since access to the clinical histories was not available to verify the patients' ethnicities, their clinical characteristics, the characterization of the type of inflammatory myopathy, its complications, its severity, disease activity, and paraclinical variables (creatine phosphokinase, antibodies, electromyography, nerve conduction velocity, images, and muscle biopsy, among others). Similarly, the medications prescribed outside the health system or not delivered by the dispensing company that the patients may have received are unknown. One strength is that this study enrolled many cases, which were distributed throughout most of the national territory, involving both the contributory and subsidized health systems of Colombia.

With these findings, we can conclude that patients with autoimmune IIMs are not treated homogeneously: the pattern of drug use varies by the type of inflammatory myopathy, by sex, by age group, by capital city versus municipality, and by system regime affiliation. Importantly, current management guidelines that include pharmacological treatment and optimal physical rehabilitation should be standardized to improve the prognosis and quality of life of IIM patients. The absence of standardized management guidelines for autoimmune myopathies impedes heterogeneous management of a pharmacological type, resulting in a lack of therapeutic adherence due to the implementation of management that can be applied for only a short time or empirically, drug changes without a comprehensive evaluation to identify an adequate clinical response, and a greater possibility of adverse drug reactions.

## Figures and Tables

**Table 1:** Comparison of some sociodemographic and pharmacological variables between the types of overlap myositis and nonoverlap myositis in Colombia.

Variables	Nonoverlap myositis		Overlap myositis		*p*
	*n* = 460	%	*n* = 211	%	
Age, median (IQR)	55.0 (41.0-65.0)		59.0 (47.0-68.0)		0.016^∗^
Women	303	65.9	173	82.0	<0.001
Comorbidities	324	70.4	211	100.0	<0.001
Arterial hypertension	154	33.5	95	45.0	0.004
Diabetes mellitus	87	18.9	52	24.6	0.089
Hypothyroidism	76	16.5	48	22.7	0.054
Rheumatoid arthritis	0	0.0	119	56.4	<0.001^∗∗^
Chronic pain	43	9.3	34	16.1	0.011
Infections	91	19.8	66	31.3	0.001
Pharmacotherapy	408	88.7	206	97.6	<0.001
Systemic glucocorticoids	349	75.9	178	84.4	0.013
Prednisolone	264	57.4	150	71.1	0.001
Prednisone	100	21.7	49	23.2	0.668
Dexamethasone	85	18.5	34	16.1	0.457
Methylprednisolone	27	5.9	25	11.8	0.007
Pulses	5	1.1	5	2.4	0.301^∗∗^
Deflazacort	29	6.3	18	8.5	0.294
Betamethasone	21	4.6	9	4.3	0.861
Hydrocortisone	8	1.7	2	0.9	0.733^∗∗^
Conventional DMARDs	303	65.9	194	91.9	<0.001
Azathioprine	205	44.6	122	57.8	0.001
Methotrexate	141	30.7	101	47.9	<0.001
Chloroquine	56	12.2	49	23.2	<0.001
Hydroxychloroquine	5	1.1	27	12.8	<0.001
Sulfasalazine	3	0.7	3	1.4	0.385^∗∗^
Leflunomide	2	0.4	3	1.4	0.182^∗∗^
Immunosuppressants	32	7.0	29	13.7	0.005
Mycophenolate	12	2.6	12	5.7	0.046
Cyclosporine	11	2.4	10	4.7	0.105
Cyclophosphamide	11	2.4	10	4.7	0.105
Human immunoglobulin	2	0.9	3	0.7	0.652^∗∗^
Biological DMARDs	9	2.0	16	7.6	<0.001
Rituximab	7	1.5	14	6.6	<0.001
Others (*n* = 4)^	2	0.4	2	0.9	0.594^∗∗^
Comedications	—	—	—	—	—
Analgesics	291	63.3	158	74.9	0.003
Antiulcer	260	56.5	153	72.5	<0.001
Antihypertensives and diuretics	188	40.9	100	47.4	0.113
Nonsteroidal anti-inflammatory drugs	186	40.4	97	46.0	0.178
Antihistamines	154	33.5	88	41.7	0.039

IQR: interquartile range; DMARD: disease-modifying antirheumatic drugs. ^∗^Mann–Whitney *U* test. ^∗∗^Fisher's exact test. ^Others: adalimumab, abatacept, belimumab, and certolizumab.

**Table 2:** Comparison of some sociodemographic and pharmacological variables between women and men diagnosed with autoimmune idiopathic inflammatory myopathies in Colombia.

Variables	Women		Men		*p*
	*n* = 476	%	*n* = 195	%	
Age, median (IQR)	53.0 (41.0-64.0)		53.0 (41.0-64.0)		0.054^∗^
Type of inflammatory myopathy	—	—	—	—	—
Overlap myositis	173	36.3	38	19.5	<0.001
Polymyositis	118	24.8	80	41.0	<0.001
Other dermatomyositis	96	20.2	49	25.1	0.156
Dermatopolymyositis	86	18.1	27	13.8	0.185
Juvenile dermatomyositis	3	0.6	1	0.5	1.000^∗∗^
Comorbidities	397	83.4	138	70.8	<0.001
Arterial hypertension	187	39.3	62	31.8	0.068
Diabetes mellitus	104	21.8	35	17.9	0.258
Hypothyroidism	96	20.2	28	14.4	0.078
Rheumatoid arthritis	97	20.4	22	11.3	0.005
Chronic pain	61	12.8	16	8.2	0.089
Infections	119	25.0	38	19.5	0.126
Pharmacotherapy	441	92.6	173	88.7	0.097
Systemic glucocorticoids	380	79.8	147	75.4	0.203
Prednisolone	305	64.1	109	55.9	0.048
Prednisone	117	24.6	32	16.4	0.021
Dexamethasone	90	18.9	29	14.9	0.214
Methylprednisolone	43	9.0	9	4.6	0.052
Pulses	9	1.9	1	0.5	0.295^∗∗^
Deflazacort	35	7.4	12	6.2	0.581
Betamethasone	22	4.6	8	4.1	0.768
Hydrocortisone	9	1.9	1	0.5	0.295^∗∗^
Conventional DMARDs	366	76.9	131	67.2	0.009
Azathioprine	238	50.0	89	45.6	0.305
Methotrexate	181	38.0	61	31.3	0.099
Chloroquine	82	17.2	23	11.8	0.079
Hydroxychloroquine	27	5.7	5	2.6	0.086
Sulfasalazine	5	1.1	1	0.5	0.678^∗∗^
Leflunomide	3	0.6	2	1.0	0.631^∗∗^
Immunosuppressants	43	9.0	18	9.2	0.936
Mycophenolate	17	3.6	7	3.6	0.991
Cyclosporine	16	3.4	5	2.6	0.590
Cyclophosphamide	15	3.2	6	3.1	0.960
Human immunoglobulin	4	0.8	1	0.5	1.000^∗∗^
Biological DMARDs	19	4.0	6	3.1	0.570
Rituximab	17	3.6	4	2.1	0.464^∗∗^
Others (*n* = 4)^	2	0.4	2	1.0	0.584^∗∗^
Comedications	—	—	—	—	—
Analgesics	328	68.9	121	62.1	0.087
Antiulcer	312	65.5	101	51.8	0.001
Antihypertensives and diuretics	216	45.4	72	36.9	0.045
Nonsteroidal anti-inflammatory drugs	204	42.9	79	40.5	0.577
Antihistamines	189	39.7	53	27.2	0.002

IQR: interquartile range; DMARD: disease-modifying antirheumatic drugs. ^∗^Mann–Whitney *U* test. ^∗∗^Fisher's exact test. ^Others: adalimumab, abatacept, belimumab, and certolizumab.

**Table 3:** Comparison of some sociodemographic and pharmacological variables between age groups of patients diagnosed with autoimmune idiopathic inflammatory myopathies in Colombia.

Variables	<65 years		≥65 years		*p*
	*n* = 491	%	*n* = 190	%	
Woman	334	69.4	142	74.7	0.173
Type of inflammatory myopathy	—	—	—	—	—
Overlap myositis	141	29.3	70	36.8	0.058
Polymyositis	136	28.3	62	32.6	0.265
Other dermatomyositis	112	23.3	33	17.4	0.093
Dermatopolymyositis	88	18.3	25	13.2	0.109
Juvenile dermatomyositis	4	0.8	0	0.0	0.582^∗^
Comorbidities	358	74.4	177	93.2	<0.001
Arterial hypertension	134	27.9	115	60.5	<0.001
Diabetes mellitus	67	13.9	72	37.9	<0.001
Hypothyroidism	65	13.5	59	31.1	<0.001
Rheumatoid arthritis	74	15.4	45	23.7	0.011
Chronic pain	49	10.2	28	14.7	0.096
Infections	107	22.2	50	26.3	0.262
Pharmacotherapy	440	91.5	174	91.6	0.966
Systemic glucocorticoids	381	79.2	146	76.8	0.501
Prednisolone	292	60.7	122	64.2	0.400
Prednisone	112	23.3	37	19.5	0.285
Dexamethasone	92	19.1	27	14.2	0.133
Methylprednisolone	40	8.3	12	6.3	0.383
Pulses	7	1.5	3	1.6	1.000^∗^
Deflazacort	34	7.1	13	6.8	0.918
Betamethasone	22	4.6	8	4.2	0.837
Hydrocortisone	7	1.5	3	1.6	1.000^∗^
Conventional DMARDs	346	71.9	151	79.5	0.045
Azathioprine	233	48.4	94	49.5	0.809
Methotrexate	166	34.5	76	40.0	0.182
Chloroquine	86	17.9	19	10.0	0.011
Hydroxychloroquine	25	5.2	7	3.7	0.407
Sulfasalazine	3	0.6	3	1.6	0.359^∗^
Leflunomide	4	0.8	1	0.5	1.000^∗^
Immunosuppressants	50	10.4	11	5.8	0.062
Mycophenolate	21	4.4	3	1.6	0.105^∗^
Cyclosporine	16	3.3	5	2.6	0.641
Cyclophosphamide	18	3.7	3	1.6	0.217^∗^
Human immunoglobulin	5	1.0	0	0.0	0.329^∗^
Biological DMARDs	21	4.4	4	2.1	0.256^∗^
Rituximab	17	3.5	4	2.1	0.462^∗^
Others (*n* = 4)^	4	0.8	0	0.0	0.582
Comedications	—	—	—	—	—
Analgesics	313	65.1	136	71.6	0.107
Antiulcer	287	59.7	126	66.3	0.111
Antihypertensives and diuretics	164	34.1	124	65.3	<0.001
Nonsteroidal anti-inflammatory drugs	215	44.7	68	35.8	0.035
Antihistamines	185	38.5	57	30.0	0.040

^∗^Fisher's exact test. DMARD: disease-modifying antirheumatic drugs. ^Others: adalimumab, abatacept, belimumab, and certolizumab.

**Table 4:** Comparison of some sociodemographic and pharmacological variables between cities and municipalities of patients diagnosed with autoimmune idiopathic inflammatory myopathies in Colombia.

Variables	Capital city		Intermediate municipality		*p*
	*n* = 508	%	*n* = 163	%	
Age, median (IQR)	56.0 (43.0-67.0)		57.0 (41.0-65.0)		0.550^∗^
Women	367	72.2	109	66.9	0.189
Type of inflammatory myopathy	—	—	—	—	—
Overlap myositis	163	32.1	48	29.4	0.528
Polymyositis	150	29.5	48	29.4	0.985
Other dermatomyositis	96	18.9	49	30.1	0.003
Dermatopolymyositis	95	18.7	18	11.0	0.023
Juvenile dermatomyositis	4	0.8	0	0.0	0.577^∗∗^
Comorbidities	410	80.7	125	76.7	0.266
Arterial hypertension	189	37.2	60	36.8	0.928
Diabetes mellitus	102	20.1	37	22.7	0.473
Hypothyroidism	100	19.7	24	14.7	0.156
Rheumatoid arthritis	93	18.3	26	16.0	0.493
Chronic pain	68	13.4	9	5.5	0.006
Infections	123	24.2	34	20.9	0.379
Pharmacotherapy	474	93.3	140	85.9	0.003
Systemic glucocorticoids	397	78.1	130	79.8	0.664
Prednisolone	312	61.4	102	62.6	0.791
Prednisone	119	23.4	30	18.4	0.180
Dexamethasone	81	15.9	38	23.3	0.032
Methylprednisolone	41	8.1	11	6.7	0.583
Pulses	7	1.4	3	1.8	0.712^∗∗^
Deflazacort	38	7.5	9	5.5	0.394
Betamethasone	17	3.3	13	8.0	0.013
Hydrocortisone	7	1.4	3	1.8	0.712^∗∗^
Conventional DMARDs	397	78.1	100	61.3	<0.001
Azathioprine	260	51.2	67	41.1	0.025
Methotrexate	195	38.4	47	28.8	0.027
Chloroquine	83	16.3	22	13.5	0.385
Hydroxychloroquine	28	5.5	4	2.5	0.139^∗∗^
Sulfasalazine	4	0.8	2	1.2	0.637^∗∗^
Leflunomide	1	0.2	4	2.5	0.014^∗∗^
Immunosuppressants	46	9.1	15	9.2	0.955
Mycophenolate	19	3.7	5	3.1	0.687
Cyclosporine	13	2.6	8	4.9	0.134
Cyclophosphamide	18	3.5	3	1.8	0.437^∗∗^
Human immunoglobulin	4	0.8	1	0.6	1.000^∗∗^
Biological DMARDs	20	3.9	5	3.1	0.610
Rituximab	16	3.1	5	3.1	0.958
Others (*n* = 4)^	4	0.8	0	0.0	0.577^∗^
Comedications	—	—	—	—	—
Analgesics	335	65.9	114	69.9	0.346
Antiulcer	312	61.4	101	62.0	0.901
Antihypertensives and diuretics	218	42.9	70	42.9	0.994
Nonsteroidal anti-inflammatory drugs	208	40.9	75	46.0	0.254
Antihistamines	183	36.0	59	36.2	0.968

IQR: interquartile range; DMARD: disease-modifying antirheumatic drugs. ^∗^Mann–Whitney *U* test. ^∗∗^Fisher's exact test. ^Others: adalimumab, abatacept, belimumab, and certolizumab.

**Table 5:** Comparison of some sociodemographic and pharmacological variables between the types of affiliation regimen to the health system of patients diagnosed with autoimmune idiopathic inflammatory myopathies in Colombia.

Variables	Contributory		Subsidized		*p*
	*n* = 590	%	*n* = 81	%	
Age, median (IQR)	58.0 (44.0-67.0)		48.0 (32.5-59.5)		<0.001^∗^
Women	415	70.3	61	75.3	0.356
Type of inflammatory myopathy	—	—	—	—	—
Overlap myositis	188	31.9	23	28.4	0.528
Polymyositis	174	29.5	24	29.6	0.980
Other dermatomyositis	124	21.0	21	25.9	0.314
Dermatopolymyositis	100	16.9	13	16.0	0.839
Juvenile dermatomyositis	4	0.7	0	0.0	1.000^∗^
Comorbidities	473	80.2	62	76.5	0.446
Arterial hypertension	230	39.0	19	23.5	0.007
Diabetes mellitus	127	21.5	12	14.8	0.162
Hypothyroidism	116	19.7	8	9.9	0.033
Rheumatoid arthritis	105	17.8	14	17.3	0.910
Chronic pain	74	12.5	3	3.7	0.015^∗∗^
Infections	141	23.9	16	19.8	0.409
Pharmacotherapy	547	92.7	67	82.7	0.002
Systemic glucocorticoids	468	79.3	59	72.8	0.183
Prednisolone	375	63.6	39	48.1	0.007
Prednisone	135	22.9	14	17.3	0.256
Dexamethasone	103	17.5	16	19.8	0.612
Methylprednisolone	45	7.6	7	8.6	0.749
Pulses	10	1.7	0	0.0	0.618^∗^
Deflazacort	40	6.8	7	8.6	0.538
Betamethasone	28	4.7	2	2.5	0.565^∗∗^
Hydrocortisone	9	1.5	1	1.2	1.000^∗∗^
Conventional DMARDs	448	75.9	49	60.5	0.003
Azathioprine	288	48.8	39	48.1	0.911
Methotrexate	219	37.1	23	28.4	0.125
Chloroquine	92	15.6	13	16.0	0.916
Hydroxychloroquine	28	4.7	4	4.9	1.000^∗∗^
Sulfasalazine	6	1.0	0	0.0	1.000^∗∗^
Leflunomide	5	0.8	0	0.0	1.000^∗∗^
Immunosuppressants	57	9.7	4	4.9	0.166^∗∗^
Mycophenolate	22	3.7	2	2.5	0.757^∗∗^
Cyclosporine	20	3.4	1	1.2	0.497^∗∗^
Cyclophosphamide	19	3.2	2	2.5	1.000^∗∗^
Human immunoglobulin	5	0.8	0	0.0	1.000^∗∗^
Biological DMARDs	22	3.7	3	3.7	1.000^∗∗^
Rituximab	18	3.1	3	3.7	0.732^∗∗^
Others (*n* = 4)^	4	0.7	0	0.0	1.000^∗∗^
Comedications	—	—	—	—	—
Analgesics	393	66.6	56	69.1	0.651
Antiulcer	363	61.5	50	61.7	0.972
Antihypertensives and diuretics	262	44.4	26	32.1	0.036
Nonsteroidal anti-inflammatory drugs	237	40.2	46	56.8	0.005
Antihistamines	211	35.8	31	38.3	0.659

IQR: interquartile range; DMARD: disease-modifying antirheumatic drugs. ^∗^Mann–Whitney *U* test. ^∗∗^Fisher's exact test. ^Others: adalimumab, abatacept, belimumab, and certolizumab.

## Data Availability

Data are available at https://doi.org/10.17504/protocols.io.b4xtqxnn.

## Additional Points

*Author Responsibility*. The corresponding author had full access to all data in the study and final responsibility. *Significance and Innovations*. Patients with autoimmune idiopathic inflammatory myopathies are not treated homogeneously. The pattern of drug use varies by the type of inflammatory myopathy, by sex, by age group, by capital city versus municipality, and by system regime affiliation. Current management guidelines that include pharmacological treatment and optimal physical rehabilitation should be standardized to improve patient prognosis and quality of life.

## Supplementary Materials

Supplementary Materials Comparison of some sociodemographic and pharmacological variables between the types of autoimmune idiopathic inflammatory myopathy in Colombia.
