# Supplementary material for: Autoimmune Idiopathic Inflammatory Myopathies: Pharmacological Differences and Similarities by Type of Myositis and by Sociodemographic Variables
Source: Int J Rheumatol. 2022 Jul 5;2022:1807571. doi: 10.1155/2022/1807571 (PMC9277175; doi:10.1155/2022/1807571)
Supplement: Supplementary Materials — Comparison of some sociodemographic and pharmacological variables between the types of autoimmune idiopathic inflammatory myopathy in Colombia. [file 1807571.f1.docx]

**Supplementary Table 1**. Comparison of some sociodemographic and pharmacological variables between the type of autoimmune idiopathic inflammatory myopathy in Colombia.

| **Variables** | **Overlap myositis** | | **Polymyositis** | | **Other dermatomyositis** | | **Dermatopolymyositis** | | **Juvenile dermatomyositis** | |
| --- | --- | --- | --- | --- | --- | --- | --- | --- | --- | --- |
|  | **n=211** | **%** | **n=198** | **%** | **n=145** | **%** | **n=113** | **%** | **n=4** | **%** |
| Age, median (IQR) | 59.0 (47.0 - 68.0) | | 59.0 (43.0 - 69.3) | | 54.0 (38.5 - 63.0) | | 52.0 (41.0 - 63.5) | | 32.0 (22.5 - 39.5) | |
| Women | 173 | 82.0 | 118 | 59.6 | 96 | 66.2 | 86 | 76.1 | 3 | 75.0 |
| Comorbidities | 211 | 100.0 | 146 | 73.7 | 95 | 65.5 | 81 | 71.7 | 2 | 50.0 |
| Arterial hypertension | 95 | 45.0 | 82 | 41.4 | 38 | 26.2 | 34 | 30.1 | 0 | 0.0 |
| Diabetes mellitus | 52 | 24.6 | 48 | 24.2 | 23 | 15.9 | 16 | 14.2 | 0 | 0.0 |
| Hypothyroidism | 48 | 22.7 | 38 | 19.2 | 14 | 9.7 | 24 | 21.2 | 0 | 0.0 |
| Rheumatoid arthritis | 119 | 56.4 | 0 | 0.0 | 0 | 0.0 | 0 | 0.0 | 0 | 0.0 |
| Chronic pain | 34 | 16.1 | 17 | 8.6 | 13 | 9.0 | 12 | 10.6 | 1 | 25.0 |
| Infections | 66 | 31.3 | 33 | 16.7 | 31 | 21.4 | 25 | 22.1 | 2 | 50.0 |
| Pharmacotherapy | 206 | 97.6 | 180 | 90.9 | 120 | 82.8 | 104 | 92.0 | 4 | 100.0 |
| Systemic glucocorticoids | 178 | 84.4 | 152 | 76.8 | 104 | 71.7 | 91 | 80.5 | 2 | 50.0 |
| Prednisolone | 150 | 71.1 | 118 | 59.6 | 72 | 49.7 | 73 | 64.6 | 1 | 25.0 |
| Prednisone | 49 | 23.2 | 47 | 23.7 | 25 | 17.2 | 28 | 24.8 | 0 | 0.0 |
| Dexamethasone | 34 | 16.1 | 27 | 13.6 | 35 | 24.1 | 21 | 18.6 | 2 | 50.0 |
| Methylprednisolone | 25 | 11.8 | 14 | 7.1 | 7 | 4.8 | 6 | 5.3 | 0 | 0.0 |
| Pulses | 5 | 2.4 | 3 | 1.5 | 2 | 1.4 | 0 | 0.0 | 0 | 0.0 |
| Deflazacort | 18 | 8.5 | 10 | 5.1 | 11 | 7.6 | 8 | 7.1 | 0 | 0.0 |
| Betamethasone | 9 | 4.3 | 8 | 4.0 | 10 | 6.9 | 3 | 2.7 | 0 | 0.0 |
| Hydrocortisone | 2 | 0.9 | 3 | 1.5 | 4 | 2.8 | 1 | 0.9 | 0 | 0.0 |
| Conventional DMARDs | 194 | 91.9 | 136 | 68.7 | 77 | 53.1 | 87 | 77.0 | 3 | 75.0 |
| Azathioprine | 122 | 57.8 | 99 | 50.0 | 46 | 31.7 | 58 | 51.3 | 2 | 50.0 |
| Methotrexate | 101 | 47.9 | 60 | 30.3 | 42 | 29.0 | 39 | 34.5 | 0 | 0.0 |
| Chloroquine | 49 | 23.2 | 13 | 6.6 | 21 | 14.5 | 21 | 18.6 | 1 | 25.0 |
| Hydroxychloroquine | 27 | 12.8 | 1 | 0.5 | 2 | 1.4 | 2 | 1.8 | 0 | 0.0 |
| Sulfasalazine | 3 | 1.4 | 1 | 0.5 | 0 | 0.0 | 2 | 1.8 | 0 | 0.0 |
| Leflunomide | 3 | 1.4 | 0 | 0.0 | 1 | 0.7 | 1 | 0.9 | 0 | 0.0 |
| Immunosuppressants | 29 | 13.7 | 13 | 6.6 | 10 | 6.9 | 9 | 8.0 | 0 | 0.0 |
| Myconefolate | 12 | 5.7 | 3 | 1.5 | 6 | 4.1 | 3 | 2.7 | 0 | 0.0 |
| Cyclosporine | 10 | 4.7 | 6 | 3.0 | 4 | 2.8 | 1 | 0.9 | 0 | 0.0 |
| Cyclophosphamide | 10 | 4.7 | 3 | 1.5 | 3 | 2.1 | 5 | 4.4 | 0 | 0.0 |
| Human immunoglobulin | 3 | 0.7 | 1 | 0.5 | 0 | 0.0 | 2 | 1.8 | 0 | 0.0 |
| Biological DMARDs | 16 | 7.6 | 5 | 2.5 | 1 | 0.7 | 3 | 2.7 | 0 | 0.0 |
| Rituximab | 14 | 6.6 | 4 | 2.0 | 0 | 0.0 | 3 | 2.7 | 0 | 0.0 |
| Others (n=4)^ | 2 | 0.9 | 1 | 0.5 | 1 | 0.7 | 0 | 0.0 | 0 | 0.0 |
| Comedications | - | - |  |  |  |  |  |  |  |  |
| Analgesics | 158 | 74.9 | 129 | 65.2 | 85 | 58.6 | 74 | 65.5 | 3 | 75.0 |
| Anti-ulcer | 153 | 72.5 | 115 | 58.1 | 73 | 50.3 | 69 | 61.1 | 3 | 75.0 |
| Antihypertensives and diuretics | 100 | 47.4 | 97 | 49.0 | 46 | 31.7 | 44 | 38.9 | 1 | 25.0 |
| Nonsteroidal anti-inflammatory drugs | 97 | 46.0 | 79 | 39.9 | 63 | 43.4 | 43 | 38.1 | 1 | 25.0 |
| Antihistamines | 88 | 41.7 | 59 | 29.8 | 57 | 39.3 | 35 | 31.0 | 3 | 75.0 |

IQR: Interquartile range. DMARD: Disease-modifying antirheumatic drugs. *Others: adalimumab, abatacept, belimumab, certolizumab.
